# The effects of atmosphere and calcined temperature on photocatalytic activity of TiO_2_ nanofibers prepared by electrospinning

**DOI:** 10.1186/1556-276X-8-548

**Published:** 2013-12-30

**Authors:** MeiLing Hu, MingHao Fang, Chao Tang, Tao Yang, ZhaoHui Huang, YanGai Liu, XiaoWen Wu, Xin Min

**Affiliations:** 1 School of Materials Science and Technology, China University of Geosciences Beijing (CUGB), 29 Xueyuan Rd. Haidian district, Beijing 100083, China

**Keywords:** TiO_2_ nanofibers, Electrospinning, Sol–gel, Photocatalytic activity

## Abstract

TiO_2_-based nanofibers were synthesized using a sol–gel method and electrospinning technique. The as-spun composite fibers were heat-treated at different temperatures (500°C, 550°C, 600°C, and 650°C) and atmospheres (ammonia and nitrogen) for 4 h. The fibers had diameters of 50 to 200 nm and mainly featured anatase and rutile phases. The anatase phase decreased and the rutile phase increased with increasing temperature. Different nitrogen conditions exerted minimal effects on the TiO_2_ crystalline phase. Different nitriding atmospheres during preservation heating yielded various effects on fibers. The effect of nitrogen in ammonia atmosphere is better than that in nitrogen atmosphere. The fibers heat-treated at 600°C and subjected to preservation heating in NH_3_ showed high photocatalytic activity.

## Background

Heterogeneous photocatalysis has been extensively investigated by researchers for the degradation of organic pollutants [1,2]. As a very promising photocatalyst, TiO_2_ shows high chemical stability, high photocatalytic activity, low cost, and non-toxicity. However, the materials exhibit photocatalytic activities only under UV light at wavelengths of less than 387.5 nm. UV light accounts for only 4% of the solar light. Therefore, synthesizing a TiO_2_ photocatalyst with visible-light responses for environmental protection is important [3-7]. The catalytic activity of TiO_2_ is easily influenced by the agglomeration of the TiO_2_ particles. TiO_2_ thin films are considered excellent photocatalytic materials because of the large specific surface area of their particles, which improves catalytic efficiency through increased contact with pollutants [8].

To improve the catalytic performance of TiO_2_ photocatalyst, researchers have investigated many methods to modify Ti. Doping with metal ions, such as the rare earth metal ions (Er, Yb, Y, and Eu) or the noble metal crystals, for example, has been performed to enhance catalytic efficiency of Ti [9-12]. However, rare metal dopant photocatalysts have low thermostability and short life spans. Furthermore, rare metals and noble metals are expensive. Several studies report that the doping of TiO_2_ with non-metals, such as carbon, nitrogen, sulfur, boron, and fluorine, shifts the optical absorption edge of TiO_2_ toward lower energies, which increases its photocatalytic activity in the visible-light region [13]. The nitrogen process is a low-cost and efficient way of modifying TiO_2_ to develop TiO_2_ fiber catalysts.

The catalytic activity of TiO_2_ is easily affected by the agglomeration of TiO_2_ particles. Thus, TiO_2_ thin films are considered as favorable photocatalytic materials. In recent years, the preparation of nanofibers by electrospinning has attracted significant research attention [14,15]. In this paper, we prepare TiO_2_ fibers by electrospinning and modify them using nitrogen at high temperatures.

### Experimental

#### Materials

The precursor for electrospinning was prepared by the sol–gel method. In a typical synthesis, 1.5 g of polyvinylpyrrolidone (PVP, molecular weight = 1,300,000) was dissolved in 20 mL of ethanol, after which 5 mL of acetic acid and 5 mL of tetrabutyl titanate were added to the above solution under magnetic stirring. After 1 h of stirring at 70°C in a water bath, the resultant orange solution was used as the electrospinning precursor.

Methylene blue (MB; concentration 20 mg/L in distilled water) was used as a model pollutant to measure photocatalytic activity of the TiO_2_ catalysts.

P25 TiO_2_ (Degussa; anatase phase, 20%; rutile phase, 80%) was used as standard photocatalytic material.

### Electrospinning

In the electrospinning procedure, the precursor solution was loaded into a 5-mL syringe with a stainless steel needle. An electric voltage of 15 kV was supplied between the needle and the collection target covered with aluminum foil. The distance between the needle and the collection target was 15 cm. A flow rate of 0.15 mm/min was supplied by a syringe pump. A white nanofiber mat was prepared by electrospinning.

PVP-Ti composite fibers were prepared by electrospinning. The as-obtained fibers were calcined at a temperature range of 500°C to 650°C at a heating rate of 1°C/min. Preservation heating was performed for 4 h under flowing N_2_ and NH_3_ surroundings.

### Characterization

The PVP-Ti composite fibers and calcined Ti fibers were characterized by various techniques such as thermogravimetry-differential scanning calorimetry (TG-DSC), x-ray diffraction (XRD), x-ray photoelectron spectroscopy (XPS), fluorescence microscopy-scanning electron microscopy (FM-SEM), transmission electron microscopy (TEM), and UV-Visible (UV–vis) spectrophotometry diffuse reflectance spectroscopy. The TG-DSC instrument was operated at a heating rate of 10°C/min in air and used to determine the thermal decomposition behavior of PVP-Ti composite fibers. Phase analysis of calcined fibers was performed using a Rigaku D/max-rA (Rigaku Corporation, Tokyo, Japan) 12 kW x-ray powder diffractometer using CuKα radiation (2*θ* = 10° to 80°). XPS spectra were recorded by a Thermo Fisher ESCALAB 250 Xi XPS instrument (Thermo Fisher Scientific, Hudson, NH, USA). The morphology and size of the calcined Ti fibers were observed by FM-SEM and TEM. UV–vis diffuse reflection spectra were used to determine the absorption spectra of the heat-treated fibers. Finally, the catalytic activity of the calcined fibers was detected by UV–vis.

### Photocatalytic experiment

The photocatalytic activity of the calcined fibers was investigated by the degradation of a standard solution of MB in a photochemical reactor. The photocatalytic reactor contained a lamp with a 500-W UV tube manufactured by Shanghai Bilon Instruments Co., Ltd. (Minhang District, Shanghai, China). About 20 mg of photocatalytic materials, including the heat-treated fibers at different temperatures and P25 TiO_2_ powders, was added into quartz tubes. About 50 mL of 20 mg/L MB solution was then added to the tubes. The mixed solutions were placed in the photocatalytic reactor, stirred in the dark for 60 min, and then exposed to UV light irradiation. UV–vis spectroscopy was used to detect the solution absorption.

## Results and discussion

### Thermoanalysis of composite fibers

**Figure 1:**
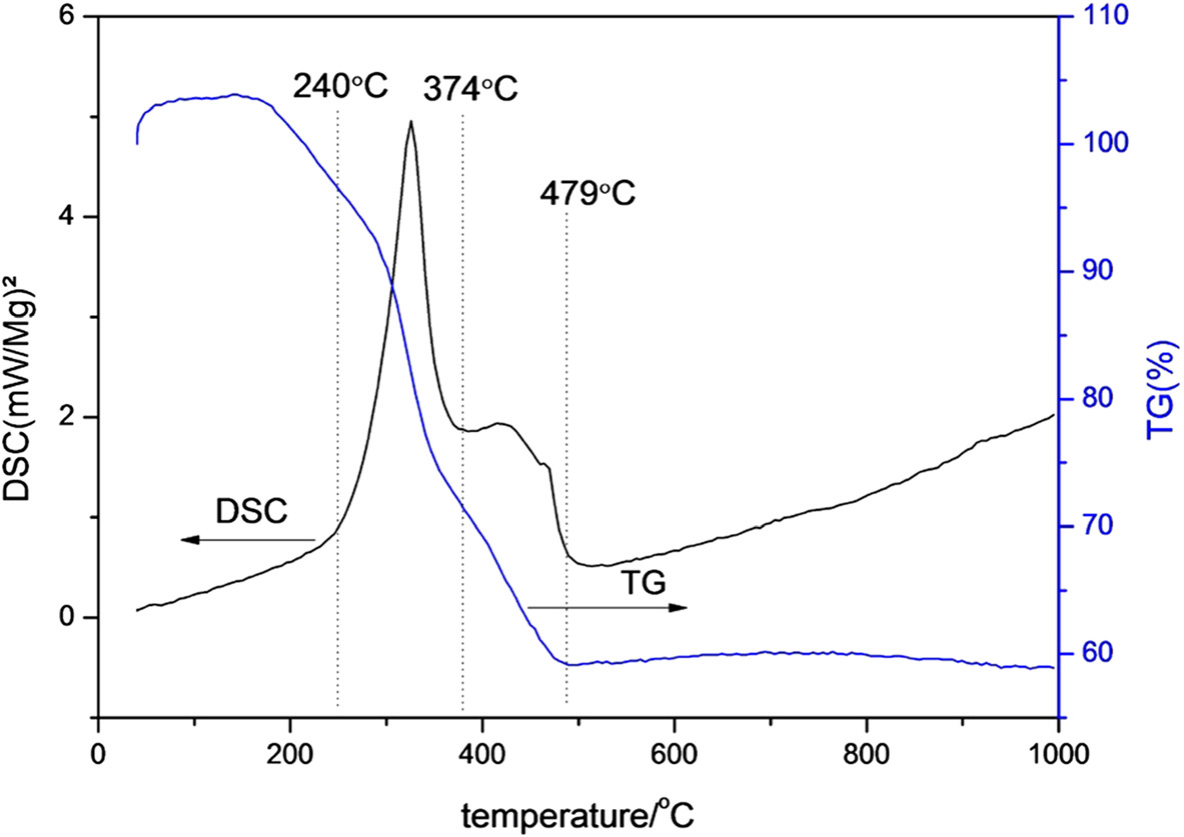
the TGA/DSC diagram for the composite fibers.

TG-DSC was performed on the PVP-Ti composite fibers mat. The curve in Figure 1 shows three weight loss stages corresponding to 240°C, 374°C, and 479°C are present. The first weight loss stage occurred below 240°C, and an endothermic band related to the DSC curve was obtained because of desorption of water and decomposition of crystal water. The rate of weight loss between 240°C and 374°C was faster than at any other temperature, and an exothermic peak attributed to the decomposition of organic components was observed. Above 479°C, no significant weight loss was observed, which indicates that the organic portion of the PVP/butyl titanate composite fibers had been completely removed. According to the DSC results from 374°C to 479°C, the curve exhibited two endothermic peaks: one from anatase structure formation and the other from phase transformation.

### Phase analysis of calcined fibers

**Figure 2:**
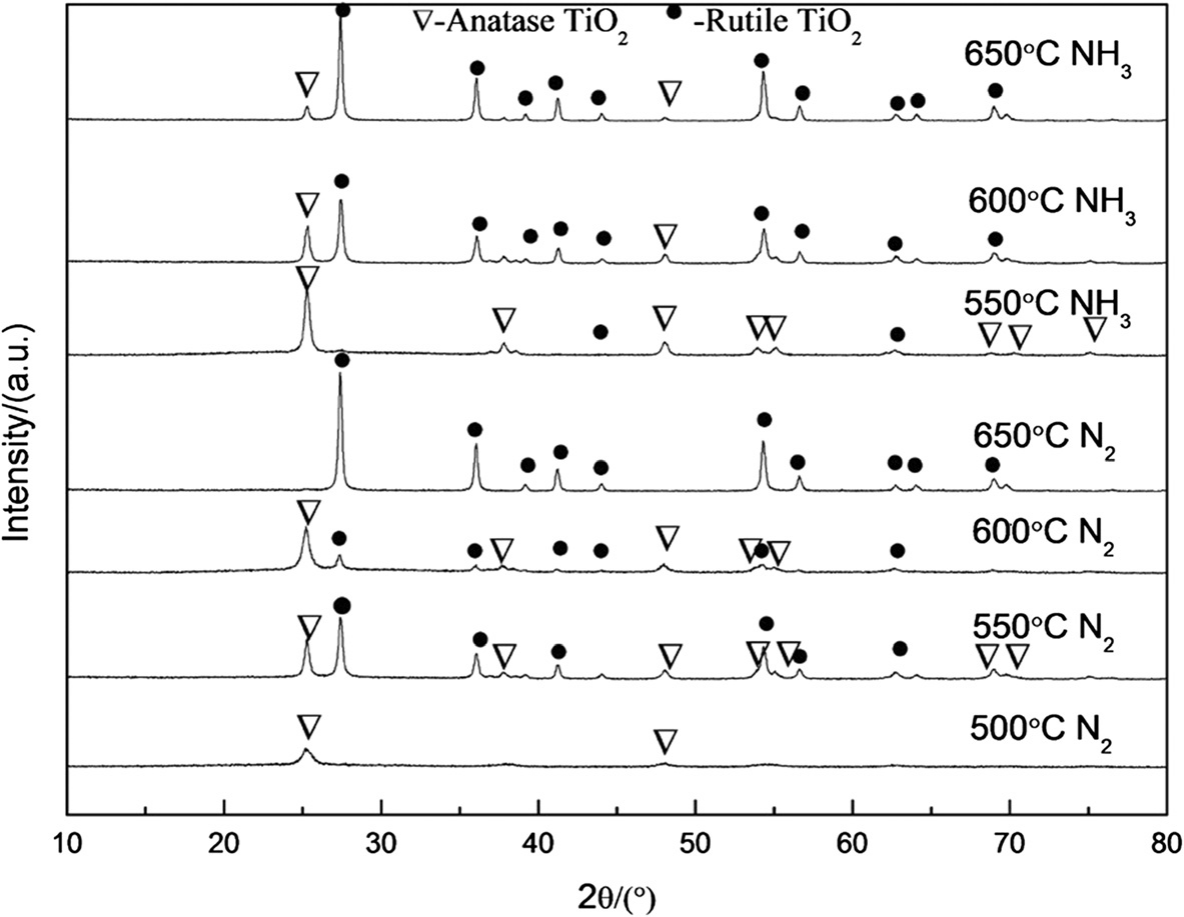
XRD patterns of composite fibers calcined in air then preserved heat in different atmospheres.

Figure 2 shows the XRD patterns of composite fibers calcined at different temperatures (500°C, 550°C, 600°C, and 650°C). After preservation in N_2_ at 500°C, a pure anatase phase was produced. The peaks of rutile phase of TiO_2_ appeared with increasing temperature. Only the pure rutile phase remained when the temperature increased to 650°C. After preservation in NH_3_ for 4 h, the samples showed a similar change process; the anatase phase with a small amount of the rutile phase appeared at 550°C. The extent of crystal transformation (from anatase phase to rutile phase) of samples under preservation heating in NH_3_ was lower than that of samples under preservation heating in N_2_. At 650°C, a small amount of anatase phase remained. A smaller degree of crystal transition was observed at this temperature because ammonia has high activity in the atmospheres, and the nitriding extent of fibers is higher than fibers in N_2_, so N atoms get into substitution position. The diffraction peak at 2*θ* = 20.9°, which corresponds to the crystalline phase of PVP, cannot be observed in the figure. These findings are consistent with the TG results, which indicate no obvious losses in the mass above 500°C [16]. According to the XRD patterns obtained, no obvious doping-related peaks appeared despite the doped samples showing characteristic TiO_2_ peaks, which may be due to the lower concentration of the doped species under nitrogen atmosphere. Moreover, the limited amount of dopants may be moved to either interstitial positions or the substitutional sites of the TiO_2_ crystal structure [13].

### Morphological analysis of calcined fibers

**Figure 3:**
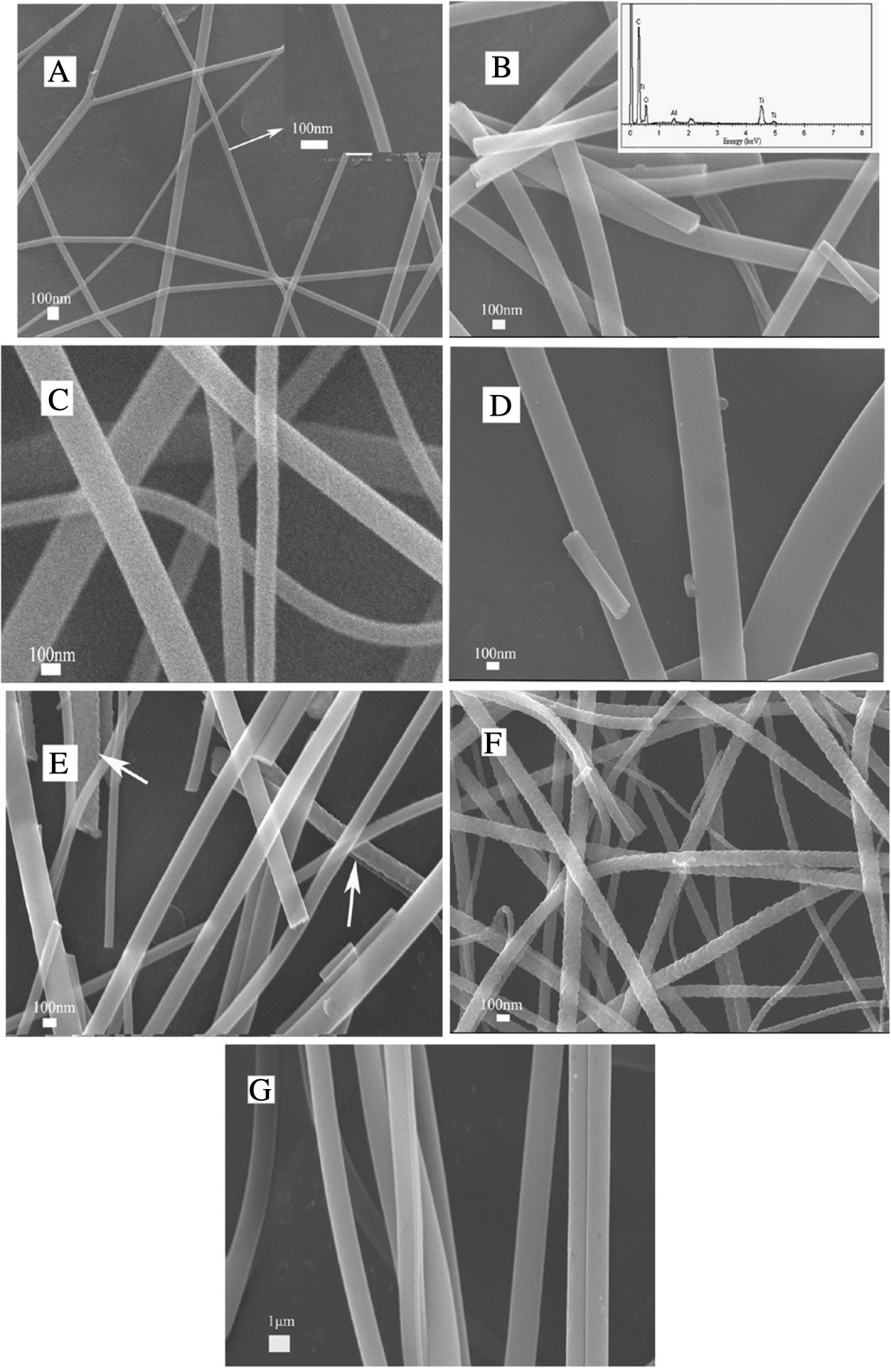
**SEM images of heat-treated electrospun fibers under different conditions. (A)** 550°C, N_2_; **(B)** 550°C, NH_3_; **(C)** 600°C, N_2_; **(D)** 600°C, NH_3_; **(E)** 650°C, N_2_; and **(F)** 650°C, NH_3_. The EDS of heat-treated fibers at 550°C in NH_3_**(G)** show the composite fibers without calcination.

Figure 3 shows the SEM images of fibers obtained under different heat-treatment conditions; fibers without calcination were also analyzed. The fibers showed smooth and homogeneous surfaces and the morphology of fibers did not change during the heating process. The average diameters of composite non-calcined and calcined fibers were approximately 500 nm to 2 μm (Figure 3G) and below 200 nm, respectively; some calcined fibers even showed diameters under 50 nm. The average diameter of calcined fibers was smaller than that of as-spun fibers because of the decomposition of organic components as the temperature increased. This result corresponds to our TG-DSC analysis. An image of the fibers calcined in N_2_ at 550°C is shown in Figure 3A. In these figures, the fiber diameter distribution was not uniform, and nanofibers with diameters of 100 ± 50 nm may be obtained. Energy dispersive spectra (EDS) results of composite fibers calcined in NH_3_ at 550°C with diameters of 200 ± 50 nm indicated the presence and relative distribution of the elements, as shown in Figure 3B. After sintering at N_2_ or NH_3_, the TiO_2_ nanofibers contained carbon but not nitrogen. The presence of carbon peaks may be attributed to the residual organics from the incomplete combustion of PVP during calcination [17,18]. The structure of fibers did not change with increasing temperature, as shown in Figure 3C,D. Figure 3E shows the composite fibers calcined in N_2_ at 650°C; some fibers were rougher than other fibers(pointed by arrow). However, the surface of the fibers obtained in NH_3_ at 650°C is rougher. This result indicates that the grain size of the fiber composites increased with increasing temperature and that ammonia promotes this process.

**Figure 4:**
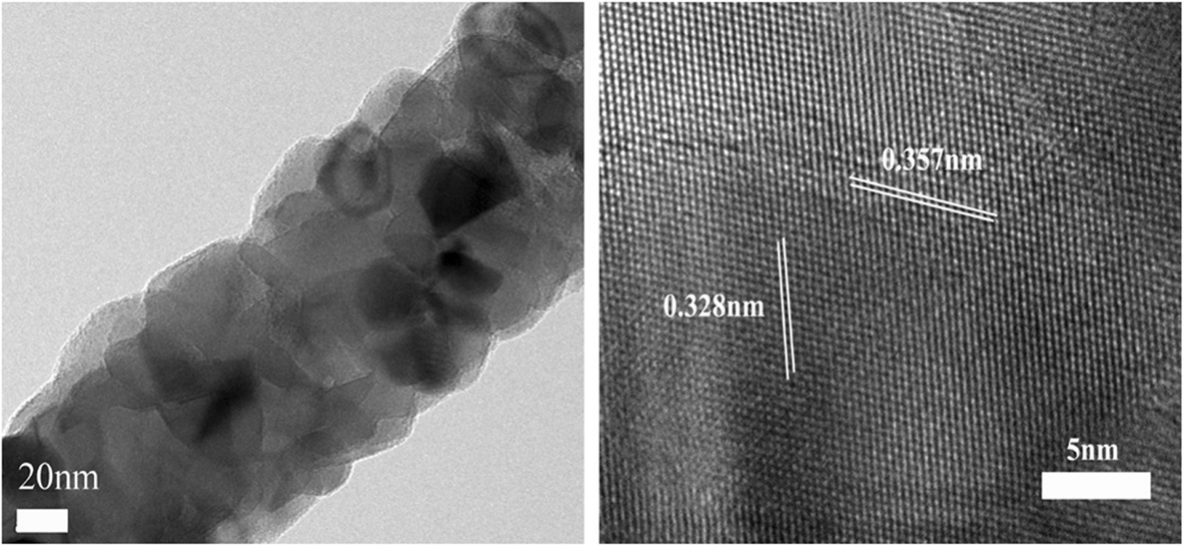
**TEM images of electrospun composite fiber calcined at 550°C then preserved heat at N**_**2 **_**for 4 h; the right image is the high-resolution TEM image.**

Figure 4 shows TEM images of an electrospun composite fiber heat-treated at 550°C and subjected to preservation heating in NH_3_ for 4 h. The low-magnification TEM image shows that the heat-treated TiO_2_ fiber has a multicrystalline structure and microcrystalline grain sizes in the range of 20 to 50 nm. The image on the right shows a high-resolution image of the TiO_2_ fiber. The lattice spacing of the crystalline structure is approximately 3.57 Å, which indicates that TiO_2_ mainly presents in anatase phase (101). The lattice spacing did not completely correspond to the standard cards; this discrepancy is believed to be due to the nitriding process adopted for preservation in N_2_ or NH_3_.

### Photocatalytic activity of calcined fibers

**Figure 5:**
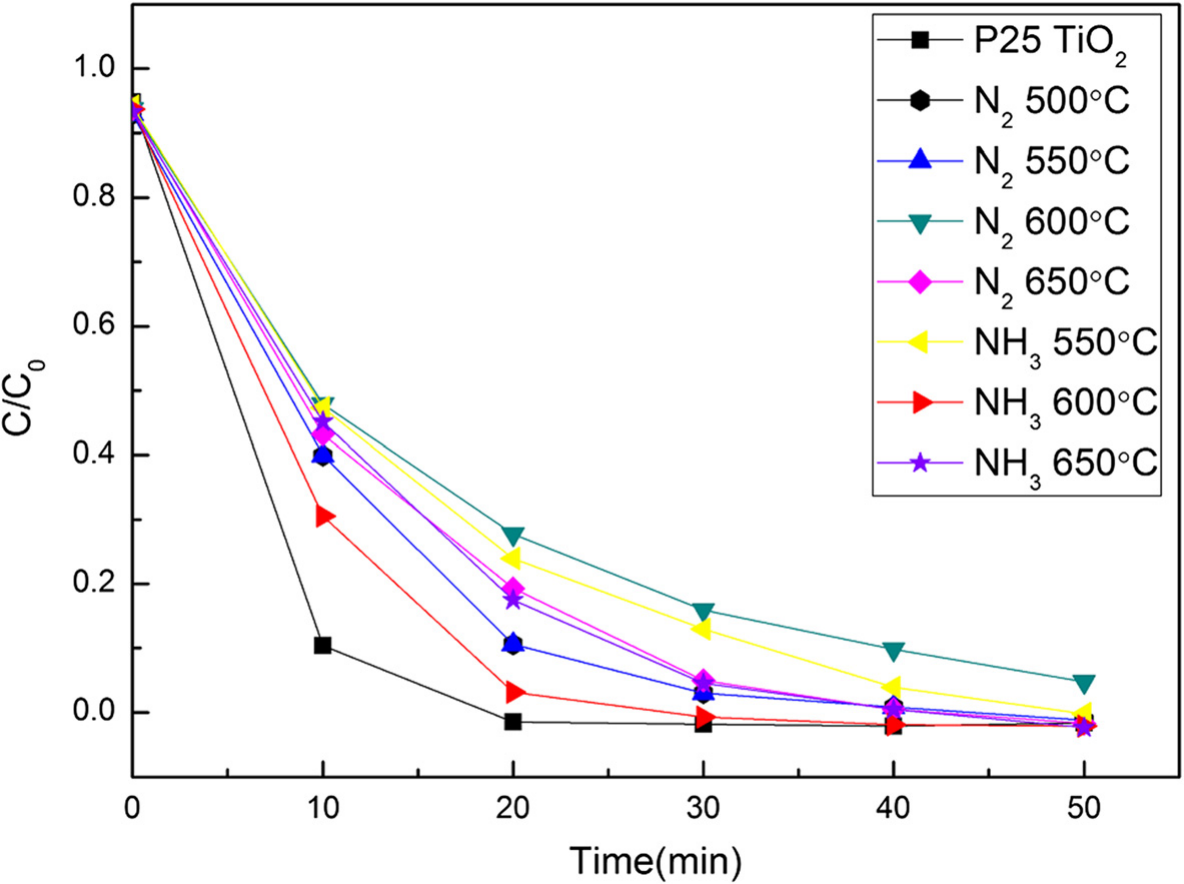
Photocatalytic activity of heat-treated fibers at different temperatures.

The photocatalysis of the samples was studied by the degradation rate of MB in UV light. P25 was used as a contrast. The samples were stirred constantly for 30 min before UV irradiation to achieve absorption equilibrium. The solutions were stirred continually under UV light irradiation, after which 5 mL of degradable MB solution was obtained every 10 min from the solutions. The samples were analyzed by UV spectrophotometry. From the results shown in Figure 5, the concentration of solution declined over 50% in the first 10 min for all fibers. After 40 min, the lowest concentration was almost below 5%. The fibers treated at 500°C and 550°C in N_2_ had the same degradation rates as the fibers treated at 650°C in N_2_ and NH_3_. This result agrees with the XRD analysis. The fibers treated at 600°C in NH_3_ showed the best catalytic activity.

**Figure 6:**
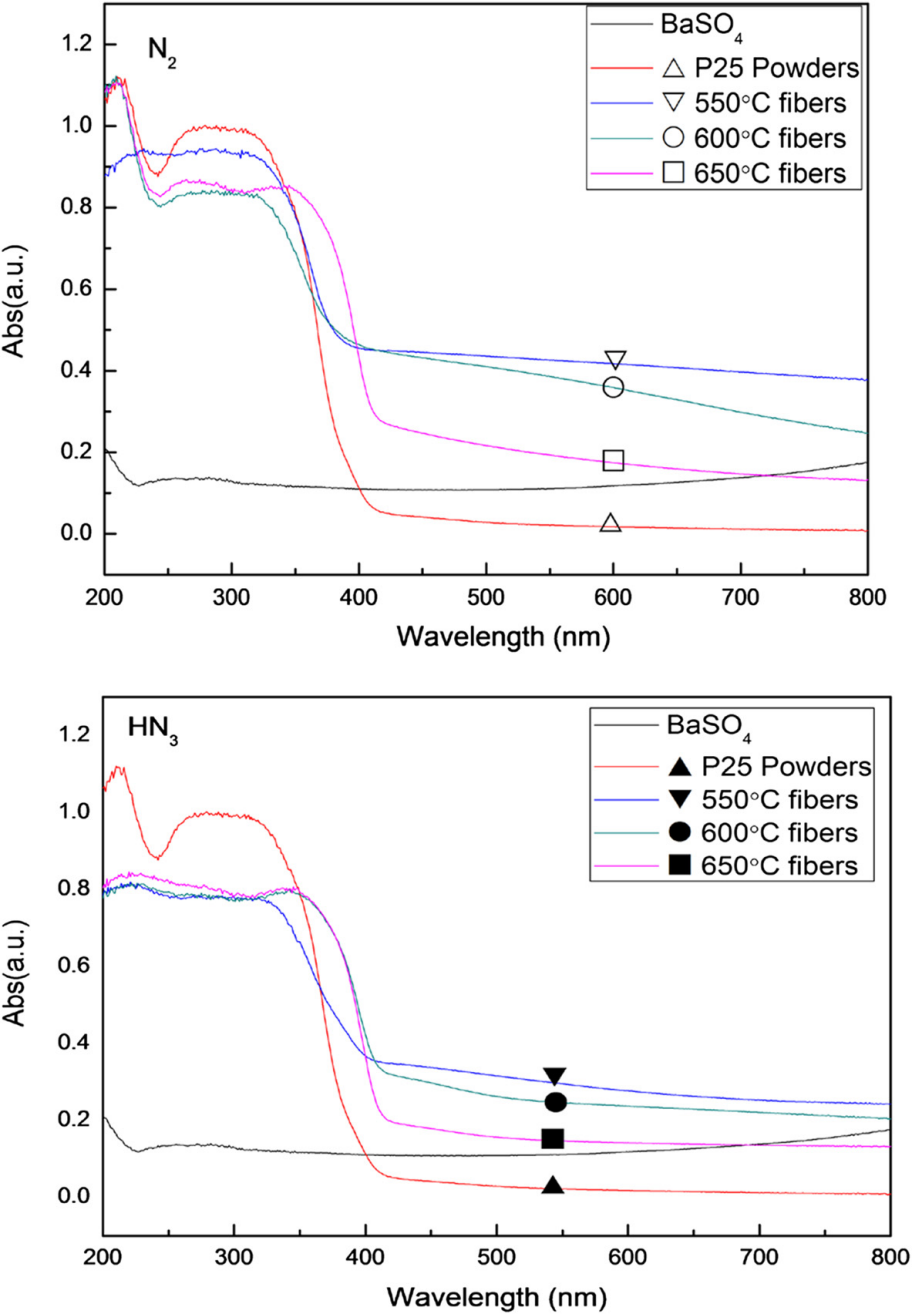
**UV–vis absorption spectra of samples at different temperatures.** UV–vis absorption spectra of samples at different temperatures in N_2_ (top) and NH_3_ (bottom) and P25 TiO_2_ powders.

Figure 6 shows the UV–vis absorption spectra of the samples that are heat-treated under different conditions as well as that of P25. The samples were heat-treated at different temperatures and then heated in N_2_ or in NH_3_ for 4 h. The curves showed strong absorption at 200 to 350 nm, which is a feature of TiO_2_. All of the fibers have different absorption strengths above 400 nm compared with P25. Above 400 nm, the absorption of P25 was nearly zero. Therefore, the synthesized fibers are responsive to visible light. Changes in the Ti-O crystalline lattice broaden the energy band by the nitriding process. At the same temperature but different protective atmospheres, the absorption strength of samples in N_2_ is stronger than that in NH_3_. The absorption strength of samples gradually decreased with increasing temperature in the same preservation atmosphere, which is caused by the transformation of the TiO_2_ crystalline phase with increasing temperature.

**Figure 7:**
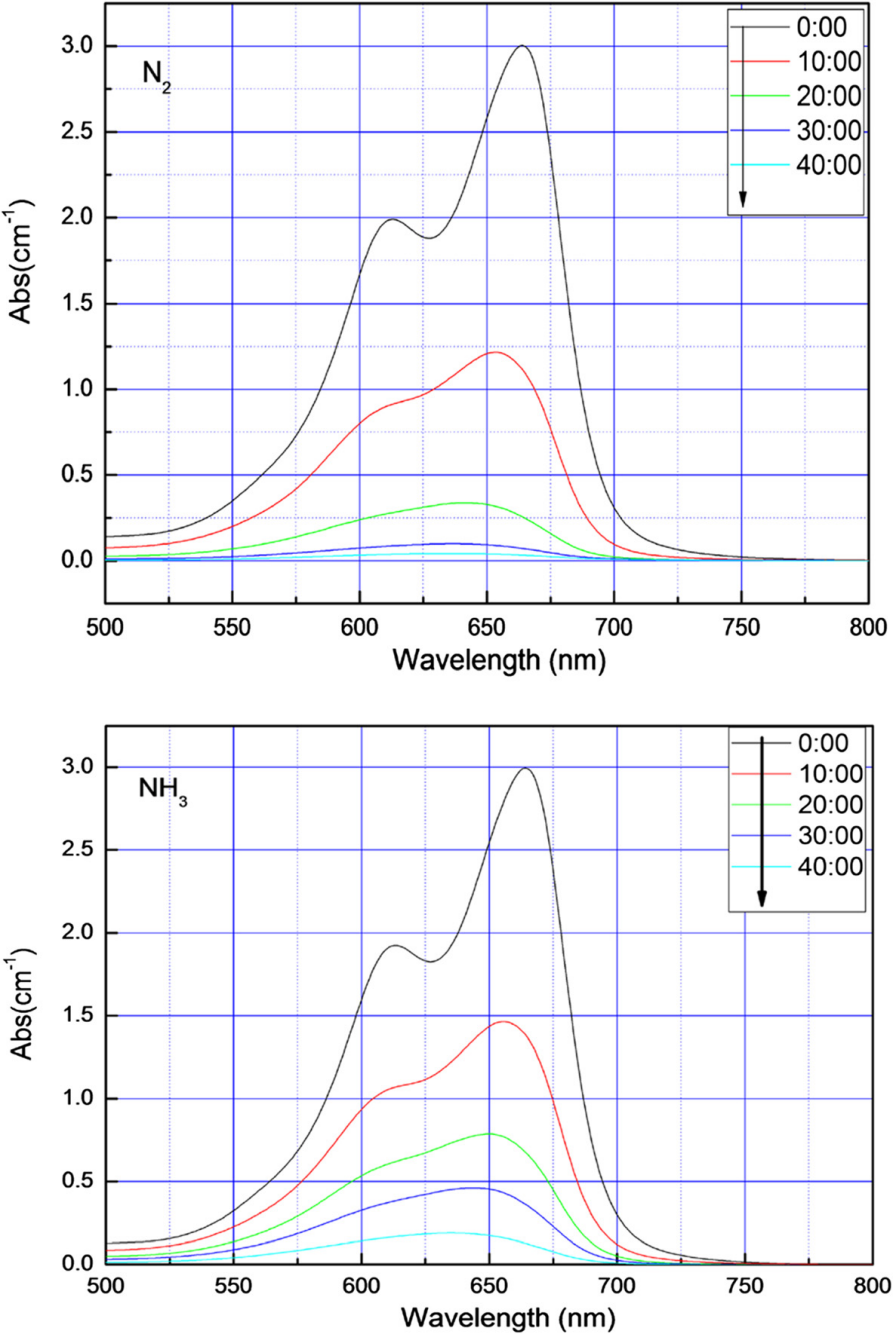
**UV–vis absorption spectra of methylene blue which were degraded by fibers.** UV–vis absorption spectra of methylene blue which were degraded by fibers at 550°C preserved heat in N_2_ (top) and NH_3_ (bottom).

Figure 7 shows the absorption spectra of the MB degraded by fibers that were heat-treated at 550°C at different atmospheres. The absorption curve has a maximum absorbance peak at 660 nm. During the experiment, the absorbance peak shifted from 660 to 645 nm after 40 min, as shown in Figure 7. According to previous researchers, reductions in the absorbance observed are probably due to the degradation of MB chromophores, and shifting of the absorption peak may be due to demethylation occurring at the catalyst surface [9,19].

### Wide-band gap analysis of calcined TiO_2_ fibers

**Figure 8:**
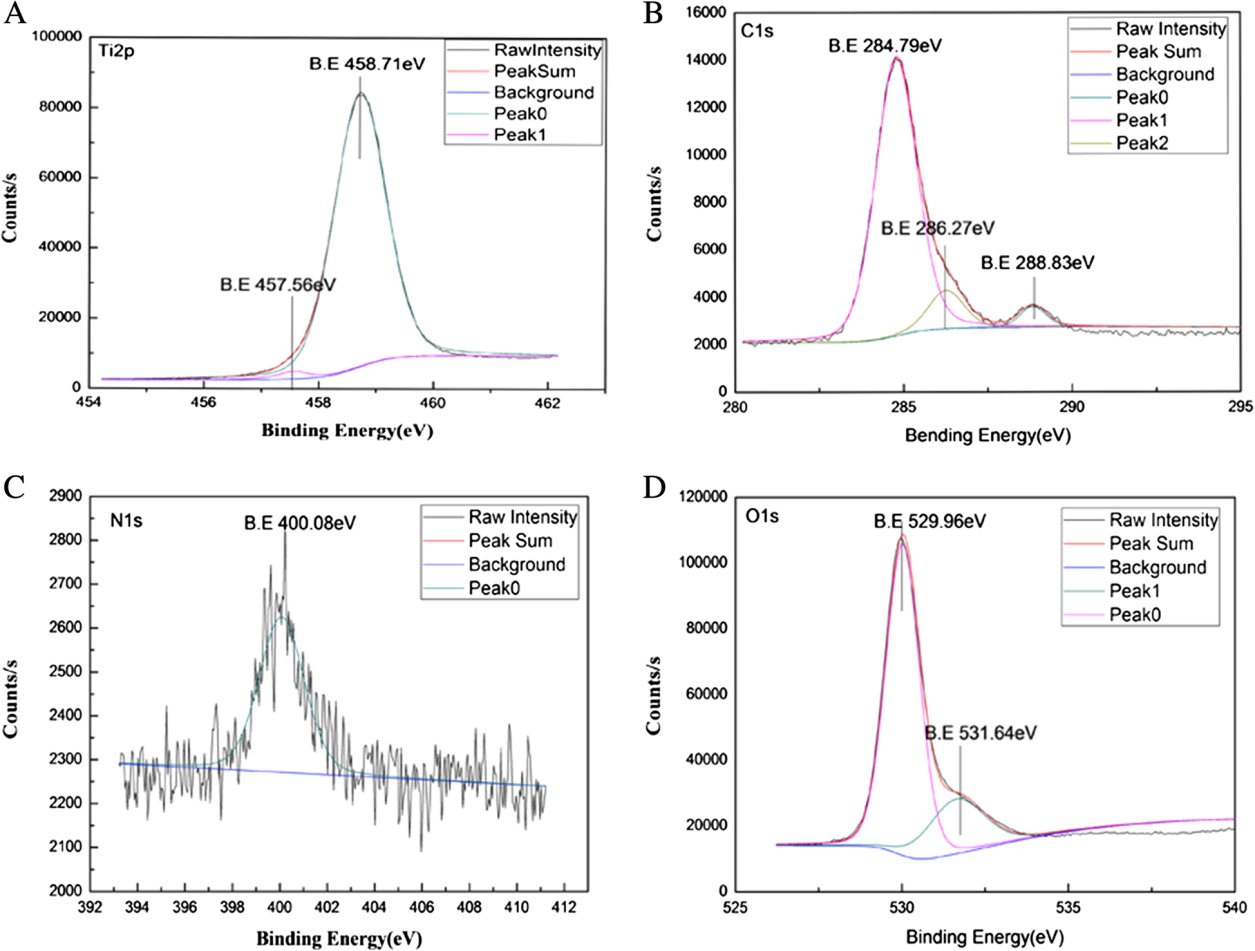
**XPS results of composite fiber heat-treated at 550°C then preserved heating in NH**_**3**_**. (A)** Ti_2*p*_, **(B)** C_1*s*_, **(C)** N_1*s*,_, and **(D)** O_1*s*_.

XPS was used to investigate the chemical status of TiO_2_ calcined at 550°C and then subjected to preservation heating in NH_3_ for 4 h. According to the XPS images in Figure 8A, the bonding energies of the Ti_2*p*1/2_ and Ti_2*p*3/2_ peaks were 458.71 and 457.56 eV, which indicates that Ti mainly exists as Ti^4+^ in TiO_2_. From the XPS spectrum of C_1*s*_ (Figure 8B), three peaks were observed at 284.79, 286.27, and 288.83 eV. The first peak was assigned to elemental carbon, which is present in the catalyst as intercalated carbon, according to previous reports [20]. The second peak of C_1*s*_ indicates that the elemental carbon exists as a C-O bond. The third peak of C_1*s*_ which indicates that elemental carbon exists as a C = O bond.

In the XPS spectrum of N_1*s*_ (Figure 8C), the dominant peak at about 400.08 eV is attributed to the adsorption of N_2_ due to surface nitriding. The surface nitriding has weakly nitrogen effects. This N element exerts no effects on the chemical status of Ti and O in the crystal lattice. Thus, the peak positions of Ti_2*p*_ and O_1*s*_ either did not change or changed only slightly. The chemistry status of N_2*p*_ did not form leading to the weak visible-light photocatalytic activity [11]. The O_1*s*_ spectra of the samples are shown in Figure 8D. The O_1*s*_ peaks of the samples were observed at 529.96 and 531.64 eV. The first peak had a binding energy of 529.96 eV, which is characteristic of metallic oxides; this result is in agreement with the O_1s_ electron binding energy arising from the Ti lattice [21,22]. In the other peak at 531.64 eV, there were several opinions to interpret the status of O_1*s*_. Emeline et al. [11] reported that the second peak is closely related to hydroxyl groups (−OH), which result mainly from chemisorbed water. The nitriding TiO_2_ may have more hydroxyl groups on its surface than pure TiO_2_. With increased surface hydroxyl content, catalysis can trap more photogenerated holes and prevent electron–hole recombination. Some studies have reported that this shift occurs mainly because of the anionic N in O-Ti-N linkages. Babu et al. [23] reported that the peak at 531.6 eV may be caused by the nitriding process changing the Ti-O crystal lattice due to the N or C doping.

## Conclusion

In summary, TiO_2_ fibers doped with non-metals (C and N) and with diameters of 100 nm were successfully produced by the electrospinning technique. The photocatalytic activity of the fibers during MB degradation was investigated after heat treatment under different atmospheres (NH_3_ and N_2_).

TG-DSC results showed that the organic groups of the composite decomposed completely at 479°C. XRD analysis showed different crystalline structures of the fibers under various heat-treatment conditions. Ti fibers containing both anatase and rutile phases showed better photocatalytic performance. SEM images showed that the diameter of the fibers ranged from 50 to 200 nm. As the temperature increased, the crystalline phases of TiO_2_ changed and exerted significant effects the nitriding process and diameter of fibers. At higher temperatures, the surface of the TiO_2_ fibers was rough, which can increase their specific surface area and improve photocatalysis. However, when the temperature was too high, TiO_2_ is given priority to trend to transform to rutile phase from anatase phase, which is detrimental for photocatalysis.

The different nitriding atmospheres of preservation heating had different effects on the fibers. The effects of nitrogen in ammonia were better than those of nitrogen because ammonia activity is higher than nitrogen activity. However, nitrogen is more economical and environment-friendly than ammonia. Heat-treated fibers at 600°C are efficient catalysts for the photocatalytic degradation of MB.

## Competing interests

The authors declare that they have no competing interests.

## Authors’ contributions

MLH, MHF, CT, TY, ZHH, YGL, and XWW independently completed this research. MLH participated in the design of the study and performed the statistical analysis and drafted the manuscript. MHF participated in its design and revised this article. CT and TY participated in a part of this experiment and the statistical analysis. ZHH, YGL, XWW and XM participated in revised this manuscript. All authors read and approved the final manuscript.

## References

[B1] Huang XH Tang YC Hu C Yu HQ Chen CS Preparation and characterization of visible-light-active nitrogen-doped TiO_2_ photocatalyst J Environ Sci 2005 8 4 562 565 16158579

[B2] Takeuchi M Matsuoka M Anpo M Hirao T Itoh N Iwamoto N Yamashita H Photocatalytic decomposition of NO under visible light irradiation on the Cr-ion-implanted TiO_2_ thin film photocatalyst Catal Lett 2000 8 2–4 135 137

[B3] Visa T Sanchez M Lopez-Grimau V Navarro R Reche S Photocatalysis with titanium dioxide to remove colour of exhausted reactive dyebaths without pH modification Desalin Water Treat 2012 8 1–3 91 99

[B4] Valencia S Cataño F Rios L Restrepo G Marín J A new kinetic model for heterogeneous photocatalysis with titanium dioxide: case of non-specific adsorption considering back reaction Appl Catal Environ 2011 8 3–4 300 304

[B5] Liu Y Liu R Liu C Luo S Yang L Sui F Teng Y Yang R Cai Q Enhanced photocatalysis on TiO_2_ nanotube arrays modified with molecularly imprinted TiO_2_ thin film J Hazard Mater 2010 8 1–3 912 20673610 10.1016/j.jhazmat.2010.07.007

[B6] Sesha SS Jeremy W Elias KS Yogi G Synergistic effects of sulfation and co-doping on the visible light photocatalysis of TiO_2_ J Alloys Compd 2006 8 1–2 322 326

[B7] Lu ZX Zhou L Zhang ZL Shi WL Xie ZX Xie HY Pang DW Shen P Cell damage induced by photocatalysis of TiO_2_ thin films Langmuir 2003 8 21 8765 8768 10.1021/la034807r

[B8] Chen C Bai H Chang C Effect of plasma processing gas composition on the nitrogen-doping status and visible light photocatalysis of TiO_2_ J Phys Chem 2007 8 42 15228 15235

[B9] Matsuo S Sakaguchi N Yamada K Matsuo T Wakita H Role in photocatalysis and coordination structure of metal ions adsorbed on titanium dioxide particles: a comparison between lanthanide and iron ions Appl Surf Sci 2004 8 1–4 233

[B10] Li Y Peng S Jiang S Lu G Li S Effect of doping TiO_2_ with alkaline-earth metal ions on its photocatalytic activity J Serbian Chem Soc 2007 8 8–9 0352 5139

[B11] Emeline AV Kuznetsov VN Rybchuk VK Serpone N Visible-light-active titania photocatalysts: the case of N-doped TiO_2s_ - properties and some fundamental issues Int J Photoenergy 2008 1 19

[B12] Li C Hou QY Zhang ZD Zhang B First-principles study on the doped concentration effect on electron lifespan and absorption spectrum of Eu-doping anatase TiO_2_ Acta Phys Sin 2012 8 7 1000 3290

[B13] Reddy PAK Reddy PVL Sharma VM Basavaraju S Kumari VD Subrahmanyam M Photocatalytic degradation of isoproturon pesticide on C, N and S doped TiO_2_ J Water Resource and Protection 2010 8 3 235 244 10.4236/jwarp.2010.23027

[B14] Wu H Pan W Lin DD Li HP Electrospining of ceramic nanofibers: fabrication, assembly and applications J Adv Cer 2012 8 2 23 10.1007/s40145-012-0002-4

[B15] Dan L Xia YN Electrospinning of nanofibers: reinventing the wheel? Adv Mater 2004 8 14 1151 1167 10.1002/adma.200400719

[B16] Alves AK Berutti FA Clemens FJ Photocatalytic activity of titania fibers obtained by electrospinning Mater Res Bull 2009 8 2 312 317 10.1016/j.materresbull.2008.06.001

[B17] Obuya EA Harrigan W Andala DM Lippens J Keane TC Jones WE Jr Photodeposited Pd nanoparticle catalysts supported on photoactivated TiO2 nanofibers J Mol Catal A Chem 2011 8 89 98 10.1016/j.molcata.2011.03.016

[B18] Kibis LS Stadnichenko AI Koscheev SV Zaikovskii SV Boronin AI Highly oxidized palladium nanoparticles comprising Pd^4+^ species: spectroscopic and structural aspects, thermal stability, and reactivity J Phys Chem C 2012 8 19342 19348 10.1021/jp305166k

[B19] Estrade-Szwarckopf H Rousseau B Photoelectron core level spectroscopy study of Cs-Graphite intercalation compounds. Clean surfaces study J Phys Chem 1992 8 3 419 436

[B20] Rizzo L Koch J Belgiorno V Anderson MA Removal of methylene blue in a photocatalytic reactor using polymethylmethacrylate supported TiO_2_ nanofilm Desalination 2007 8 1 9 10.1016/j.desal.2006.02.081

[B21] Yang QL Sun Y Su JX Su J Guo L Jiang L Preparation of visible-light active N-doped nano-TiO_2_ photocatalyst by hydrothermal method Identify Applicable Sponsor 2011 8 1433 1438

[B22] Rane KS Mhalsiker R Yin S Sato T Cho K Dunbar E Biswas P Visible light-sensitive yellow TiO_2-x_N_x_ and Fe–N co-doped Ti_1-y_Fe_y_ O_2-x_N_x_ anatase photocatalysts J Solid State Chem 2006 8 3033 3044 10.1016/j.jssc.2006.05.033

[B23] Babu JV Rao PR Sreekumaran AN Nitrogen-doped rice grain-shaped titanium dioxide nanostructures by electrospinning: frequency and temperature dependent conductivity J Appl Phys 2011 8 6 064327 064333 10.1063/1.3642979

