# Erratum to: The effects of atmosphere and calcined temperature on photocatalytic activity of TiO_2_ nanofibers prepared by electrospinning

**DOI:** 10.1186/s11671-015-0840-4

**Published:** 2015-04-11

**Authors:** MeiLing Hu, MingHao Fang, Chao Tang, Tao Yang, ZhaoHui Huang, YanGai Liu, XiaoWen Wu, Xin Min

**Affiliations:** School of Materials Science and Technology, China University of Geosciences (Beijing), 29 Xueyuan Rd. Haidian district, Beijing, 100083 China

## Erratum

Unfortunately, the address of the corresponding author was presented inaccurately in the published version this paper [1]. Instead of China University of Geosciences Beijing (CUGB), it should be China University of Geosciences (Beijing).
